# Municipal community centers as healthy settings: evaluation of a real-world health promotion intervention in Jerusalem

**DOI:** 10.1186/s12889-022-14220-7

**Published:** 2022-10-07

**Authors:** Deborah Barasche-Berdah, Sima Wetzler, Iva Greenshtein, Keren L. Greenberg, Elisheva Leiter, Milka Donchin, Donna R. Zwas

**Affiliations:** 1 The Linda Joy Pollin Cardiovascular Wellness Center for Women, Hadassah University Medical Center, Ein Kerem, P.O.B. 12000, 91120 Jerusalem, Israel; 2 Israel Healthy Cities Network and Braun School of Public Health and Community Medicine, Hebrew University, Jerusalem, Israel

**Keywords:** Health promotion, Settings, Healthy communities, Community centers, Evaluation strategy, RE-AIM framework, EQUIHP

## Abstract

**Background:**

This study presents an intervention designed to foster the implementation of health promotion programs within District Municipality Community Centers (DMCCs) in Jerusalem, and the creation of a peer network of healthy settings with a shared aspiration of collaborating and implementing health-promoting policies at the community level. We also present the evaluation strategy, based on the EQUIHP and RE-AIM frameworks.

**Methods:**

Twenty DMCCs completed our program. This evaluation research involved a comprehensive seminar during the first year for DMCCs coordinators, teaching them the principles of health promotion. An educational kit was distributed during the second year. The evaluation strategy included a process evaluation and annual evaluations based on the EQUIHP and RE-AIM frameworks. The EQUIHP tool was divided into four dimensions of evaluation: 1) Framework of health promotion principles, 2) Project development and implementation, 3) Project management, and 4) Sustainability; while the RE-AIM domains included: 1)Reach, 2)Effectiveness, 3)Adoption, 4)Implementation and 5)Maintenance.

**Results:**

The program led to high responsiveness among DMCCs and to the implementation of diverse health promotion initiatives, with a participation of approximately 29,191 residents. The EQUIHP evaluation showed an improvement in program quality in Year 2*.* The final RE-AIM evaluation presented a total median score of 0.61 for all domains, where 0 was non-performance and 1.0 was full performance. The ‘Framework of health promotion principles’ and ‘Reach’ components received the highest median score (0.83, 1.0 and 0.87), while the ‘Sustainability and ‘Maintenance’ components received the lowest (0.5).

**Conclusions:**

This innovative program adapts the Healthy Cities approach (initiated by the World Health Organization in 1986) to the development of community center health-promoting settings within the larger municipal framework, training local community center staff members to assess and address local health concerns and build community capacity. The local focus and efforts may help community actors to create health promotion programs more likely to be adopted, feasible in the ‘real-world’ and able to produce public health impact in the communities where people live. Moreover, collaboration and cooperation among DMCCs may lead to a broader community health vision, forging coalitions that can advocate more powerfully for health promotion.

**Trial registration:**

NIH trial registration number: NCT04470960.

Retrospectively registered on: 14/07/2020.

**Supplementary Information:**

The online version contains supplementary material available at 10.1186/s12889-022-14220-7.

## Background

The Ottawa Charter encourages the use of “healthy settings” in health promotion (HP), as these are natural environments where people spend most of their time [1]. Global urbanization strengthens the need for municipal community action promoting health in cities. The Healthy Cities movement [2], initiated by the World Health Organization (WHO) European Office in 1986, has become a leading mechanism for integration of HP within municipalities, enabling synergistic interactions between governmental and non-governmental entities, as well as sharing of ideas and resources between municipalities. The cross-disciplinary collaboration among public health workers and city planners also allows for more effective HP in urban contexts.

Application of the Healthy Cities model to municipal community centers constituted the rationale behind this study as this model may enhance population health, tailoring interventions to cultural and local needs. These community centers may then become an important foundation for HP with the potential to directly influence the population. Community-based interventions at the municipal and neighborhood level can benefit from the interaction between the built environment, cultural and social assets as well as residents’ specific health needs and strengths. Despite these potential benefits, a city-wide evaluation of health promotion programs (HPPs) conducted in Jerusalem found that municipality-sponsored programs received the lowest scores on program quality compared to non-governmental or health organizations [3], which justified action in the municipality sphere.

Jerusalem is the largest and most populous city in Israel, approaching one million residents [4]. The population is highly heterogeneous, with marked variation of socio-economic status and ethnic and religious identification. This led to the creation of a unique governing structure whereby district municipal community centers (DMCCs) provide municipal and community center services “all in one”. The District Municipal Community Center (DMCC) is an independent legal entity, administered by the Community Centers Association [5] and the Jerusalem Municipality, with officials elected by local residents. The DMCC represents all neighborhood matters to the municipality and is responsible for urban planning and community development within its geographic jurisdiction. The DMCC is responsible for the provision of services to the residents of that district. As part of its activities, the DMCC conducts cultural, enrichment, and leisure programs, as well as fostering neighborhood coalitions and community dialogue between different population groups. Goals include maximizing self-direction for residents, promoting a high quality of life, and increasing residents’ involvement in volunteer activities for the neighborhood. The municipality determines the budget, together with the DMCC administration itself.

This study presents an intervention aimed to foster the establishment and expansion of HPPs conducted by DMCCs so as to reduce health disparities and to create a peer network of healthy settings with a shared aspiration of collaborating and implementing health-promoting policies at the community level. We also present the evaluation strategy, based on the EQUIHP tool [6] and RE-AIM framework [7].

## Methods

### Program overview of this evaluation research

The health-promoting community center (HPCC) program was conducted by the “Jerusalemites Choose Health” (JCH) program, a joint effort of the Jerusalem Municipality, the Ministry of Health and the Jerusalem District Health Bureau. The JCH further collaborated with additional offices within the Jerusalem Municipality, including the Jerusalem Municipality Sports Authority, the Jerusalem Health Education and Promotion Department, and the Linda Joy Pollin Cardiovascular Wellness Center for Women at Hadassah University Medical Center. The Pollin Center was asked to evaluate the program.

Criteria for a HPCC were established by consensus and incorporated into an agreement signed by participating DMCCs (see Additional file 1, Appendix A).

It was hypothesized that DMCCs who will receive the intervention will have better skills at building sustainable participatory effective HP initiatives and will provide a healthy setting for the community within their jurisdiction. Thus, the study questions prior to this evaluation research were ‘to what extend the intervention process and components will increase knowledge of DMCCs’ staff members in establishing HPPs?’ and ‘how this intervention will foster the planning and implementation of HPPs according to the HPCC criteria, as evaluated by the EQUIHP and RE-AIM frameworks?’.

**Fig. 1:**
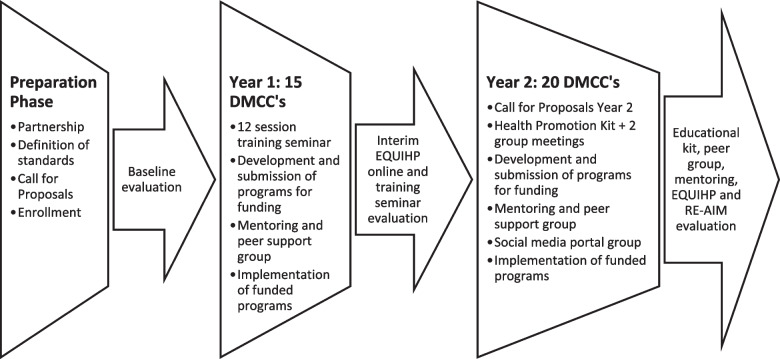
Program Timeline and description of the preparation phase, first- and second-year activities

DMCCs were invited to participate in this program via a call for proposals circulated to all 31 DMCCs in Jerusalem and re-issued after the first year of the program. The invitation included four pre-requisites:1) the DMCC director was requested to sign a letter of intent to define their center as a health promoting setting and to act in accordance with the declaration, 2) assignment of a HP coordinator who will participate in a 12 session HP course, 3) submission of a questionnaire regarding HP policy and actions being done within the community center and the neighborhood surrounding it, 4) sending an attached letter explaining the reason the DMCC is interested in joining the project and elaborating on actions currently being done by the DMCC in specific HP areas. These pre-requisites were the inclusion criteria for participating in the program. Funding was made available for HPPs that met criteria and included goals, objectives, activities, a budget and a plan for evaluation. The call for proposals was re-issued to all DMCCs after the completion of the first year of the program, without the requirement for participation in the seminar. The structure of the program is presented in Fig. 1.

#### Training seminar

During Year 1, all health coordinators, one from each participating DMCC, were required to attend a twelve-session weekly training seminar. Each session lasted 5 h. The seminar coordinator was a community project manager from the Pollin center and a trained researcher in the HP field. She was one of the speakers and moderator during panel discussions and workshop sessions. Additional professional speakers delivered presentations and workshops during this seminar: public health doctors, HP specialist from the Ministry of Health, physiotherapist, nutritionist, etc. The seminar taught the principles of HP, and trained and supervised coordinators in the knowledge, skills and methods for planning and implementing HPPs. They learned about the standards of what is a HPCC. The themes delivered during this seminar included basic knowledge in nutrition, physical activity, smoking and healthy environments. They also learned about leadership, planning principles, writing skills, implementation of an annual HP program, and building internal and external partnerships. Participating coordinators were trained and guided to perform health needs assessment in their community and to write an annual work plan. The training seminar also encouraged and enabled the creation of a peer-network of HPCCs. In the final class, each coordinator presented his/her HP program work plan. See Supplemental Table 1 for the curriculum outline.

#### Educational kit

In Year 2, a HP online resource kit was distributed for coordinators’ self-education and program planning. The kit included HP materials, ideas for HP evidence-based initiatives, and manuals for activities with various target populations (children, elderly, families, immigrants, etc.). Topics included healthy workplaces, health-promoting settings, HPPs developed by the ministries of education and health and lists of potential partners and service providers. Coordinators were encouraged to use these materials for self-education and program-planning. The new coordinators, who joined in Year 2, received one-on-one mentoring and telephone follow-up (as they had not participated in the group seminar). During April 2019, each coordinator presented their HPPs work plan at a formal group meeting and received feedback based on HP practices by the program steering committee and leading advisors.

#### Peer group and mentoring

To facilitate the creation of an ongoing peer network, a social media portal group discussion was created including the research team. This provided a platform wherein coordinators could discuss challenges, problem-solve and share successes. Trained researchers mentored coordinators via monthly phone calls. They discussed programs implemented, challenges and issues that required guidance or assistance.

### Evaluation

#### Baseline and training seminar

Questionnaires were filled out by each DMCC, assessing the baseline characteristics of the community center and coordinators.

Furthermore, at the end of the course, seminar attendees filled out an anonymous questionnaire addressing participants’ knowledge, skills and health behaviors.

#### Educational kit, peer group and mentoring

A process evaluation for assessing kit use and the social media portal group participation was performed at the end of year 2. Participants were asked the following questions: “Have you used this kit? What materials were the most useful for you? What would you suggest adding to this kit?”

The research team recorded details on HP initiatives, new partnerships, challenges and mentoring concerns through the monthly mentoring phone calls.

#### EQUIHP evaluation

At the end of Year 1 and during Year 2, coordinators assessed each HP activity using an online 28-item tool, adapted from the European Quality Instrument for Health Promotion (EQUIHP) [8] that was culturally adapted and translated. The EQUIHP tool is usually used to assess HPPs, for quality improvement or as a checklist for self-evaluation [6]. The tool is divided into four dimensions, considering the important factors for effective HP: 1) Framework of HP principles, 2) Project development and implementation, 3) Project management, and 4) Sustainability.

#### Final RE-AIM evaluation

For a more complete and detailed assessment, the final evaluation at the end of Year 2 was based on the RE-AIM framework [7], an accepted and robust model in the public health domain, used for planning, implementing, and evaluating several types of HP interventions. Dimensions include reach(R), effectiveness(E), adoption(A), implementation(I) and maintenance(M) [9]. The RE-AIM framework has been used for reporting internal and external validity [10, 11]. The different measures for evaluating and measuring inclusion of RE-AIM components are presented in Table 1. Relevant elements of the EQUIHP tool [8] were also incorporated to the finalized RE-AIM tool by an expert committee, resulting in a final 29-item tool. Our RE-AIM evaluation investigated each DMCC and evaluated adherence to HP principles and practices in the proposed work plans and projects implementation.

**Table 1:** Evaluation within RE-AIM dimensions

RE-AIM dimensions and original definitions (Glaskow, 1999)	Related Items and pragmatic use of RE-AIM evaluation in the current study
REACH Number, proportion and representativeness of the target population	Inclusion of various target populations
	Reach of the DMCCs employees
EFFECTIVENESS The impact of an intervention on outcomes/ success rates	Use of best practice strategies and evidence-based practice
	Inclusion of needs assessment in planning process
	SMART objectives
	Adaptation to the environmental context
	Monitoring, process and outcome evaluation
	Defined goal
ADOPTION Proportion of settings, practices, and plans that will adopt this intervention	Steering committee for the program
	Representation of community members and DMCC director in the steering committee
	Involvement and support of the DMCC director for integrating the program into practice
	Financial assistance
IMPLEMENTATION Extent to which the intervention is implemented as intended in the real world	Extent to which the program is implemented according to plan
	Description of the implementation strategy
	Type of intervention and intensity
	Coping efforts in face of challenges
	Creation of a peer-network
MAINTENANCE Extent to which a program is sustained over time	Policy and/or practice changes regarding health promotion
	Duration and sustainability of programs
	Meeting criteria for HPCC

### Data analysis

For summarizing and describing our results, the characteristics of the data were analyzed using descriptive statistics. Descriptive coefficients regarding the frequency distribution, central tendency, and variability of the dataset were assessed. For ordinal data, median and interquartile range (IQR) were used [12]. The data is presented in a graphical way, providing a useful visual summary of the results. The box plots enabled the researchers to quickly identify and compare the dispersion of the data set. The evaluation data were analyzed using IBM SPSS statistics 25 (Chicago, IL, USA).

For baseline and training seminar evaluation, frequencies were calculated to describe and quantify the questionnaire variables.

For evaluation of the educational kit, peer group and mentoring, a researcher organized and summarized the data by frequencies calculation and qualitative analysis. The content of the social media portal discussions was assessed by thematic analysis.

For EQUIHP evaluation, each item of the adapted tool was coded online by the coordinators, according to three options of replies: not relevant or absent, partially present, or present, adhering to EQUIHP instructions. Assistance in reporting and coding was provided by the research team to coordinators as necessary. Two items regarding the planned and actual number of participants were open questions and were categorized by a researcher. Then, to obtain a comparative index and based on a previous study [13], each item was recoded on a 0–1 scale and according to the following rules: (0 = not done), (0.5 = partially complete), (1 = complete). The sum of valid scores was divided by the number of valid answers. Absolute and median scores on a 0–1 scale were calculated for each EQUIHP dimension of the reported HP initiatives.

For the final RE-AIM evaluation, to improve the accuracy of data collection, data was collected via in-depth telephone interviews (rather than online) at the end of Year 2, coded and interpreted by the research team. The in-depth interviews were intended to avoid possible bias from the online reports and subjective self-coding by coordinators. After transcription of all interviews by a trained researcher, the data were coded and interpreted by the research team only. First, by one researcher separately, then to ensure accuracy and reduce bias, two additional independent researchers reviewed and discussed coding and conclusions. Each item was coded on a 0–1 scale (0 = not relevant or absent, 0.5 = partially present, 1 = present). Absolute and median scores were calculated for each DMCC and RE-AIM components.

## Results

In Year 1 (2018), 16 DMCCs applied (completed the application and questionnaire) and 15 fulfilled the requirements for inclusion. One DMCC was excluded due to the fact that staffing changes led to the inability to appoint a coordinator to participate in the training seminar and administer the program. In Year 2 (2019), 7 more DMCCs joined, for a total of 22 DMCCs out of a possible 31, but two subsequently dropped out, also due to inability to identify a coordinator. Twenty DMCCs completed the program at the end of 2019. A written annual work plan of the planned HP activities was provided by 84% of DMCCs. All the participating DMCCs reported on their health initiatives and activities during the evaluation phase.

### Baseline and training seminar

The characteristics of participating DMCCs and coordinators are presented in Table 2. The DMCCs most often served mid-sized populations (15,000–30,000 residents). Fifteen DMCCs participated in both the first and second year of the program. Of the fifteen, 8 DMCCs had a different coordinator responsible for the program in the second year. The targeted populations of the programs were designated as men and women, families, children and teenagers, older individuals or DMCC employees.

**Table 2:** Characteristics of participating DMCCs and coordinators

**DMCC's (*n* = 20) variables**	
*Community size*	
<15,000	17%
15,000−30,000	44%
>30,000	39%
*Target populations of programs*	
Women only	12%
Men only	4%
Parents	9%
Children and preschoolers	15%
Teenagers	10%
Elderly	6%
Staff	9%
'Open to all' event	30%
Population of special needs	5%
*Health promotion program written annual work plan*	
Yes	84%
No	16%
*Participation in the Network/program*	
DMCC 2 years	75%
DMCC only in year 2	25%
Coordinator turnover after year 1	53%
*Health promotion*	
Comprehensive health promotion activity	26%
Nutrition only	20%
Physical activity only	37%
Other	16%
*Coordinators Variables*	
*Education*	
Nonacademic level	32%
BA level	50%
MA and higher level	18%

The training seminar’s post questionnaire results are presented in Table 3. All the coordinators indicated that the seminar increased their knowledge about HP (84.6% strongly agreed and 15.4% somewhat agreed) and health-promoting settings (76.9% strongly agreed, 23.1% somewhat agreed). They all reported receiving support from their municipality director throughout their participation in the seminar (92.3% strongly agreed, 7.7% somewhat agreed) and were interested in new collaborations and networking with the local Health Maintenance Organizations (that provide health insurance and medical services to Israeli citizens), (91.7% strongly agreed, 8.3% somewhat agreed). At the end of the seminar, all the coordinators declared interest in initiating health policy changes in their community center (83.3% strongly agreed, 16.7% somewhat agreed) and most of them reported that they acquired practical tools for this purpose (76.9% strongly agreed, 15.4% somewhat agreed and 7.7% somewhat disagreed).

The training seminar was less likely to affect personal health habits, such as physical activity (only 23.1% strongly agreed) or diet (15.4% strongly agreed, 15.4% somewhat agreed).

**Table 3:** Training seminar process evaluation results

Items	Strongly Agree (%)	Somewhat Agree (%)	Somewhat Disagree (%)	Strongly Disagree (%)
The seminar added to my knowledge- nutrition	46.2	7.7	38.5	7.7
The seminar added to my knowledge- physical activity	30.8	15.4	46.2	7.7
The seminar added to my knowledge- smoking	53.8	23.1	15.4	7.7
The seminar added to my knowledge- the elderly population	30.8	61.5	7.7	0.0
The seminar added to my knowledge- the special needs population	7.7	46.2	46.2	0.0
The seminar added to my knowledge- the infant and toddler population	25.0	25.0	41.7	8.3
The seminar added to my knowledge- health promotion	84.6	15.4	0.0	0.0
The seminar added to my knowledge- health-promoting settings	76.9	23.1	0.0	0.0
The seminar added to my knowledge- programs evaluation	41.7	25.0	33.0	0.0
The seminar added to my knowledge- community work	16.7	25.0	41.7	16.7
I developed in the seminar new connections that will help me in my current work in the community center	58.3	33.3	8.3	0.0
I would love to participate to an additional seminar in the future	53.8	15.4	15.4	15.4
I had the support of my director throughout the seminar	92.3	7.7	0.0	0.0
Thanks to the seminar, I am interested in creating new collaborations	91.7	8.3	0.0	0.0
Thanks to the seminar, I am interested in working with the health maintenance organizations’ representatives	91.7	8.3	0.0	0.0
Thanks to the seminar, I eat a more balanced diet	15.4	15.4	38.5	30.8
Thanks to the seminar, I am more active	23.1	0.0	23.1	53.8
Thanks to the seminar, I am more aware of the physical environment in my community center	46.2	7.7	38.5	7.7
Thanks to the seminar, I am interested in initiating health policy changes in my community center	83.3	16.7	0.0	0.0
Thanks to the seminar, I acquired tools for health promotion in my community center	76.9	15.4	7.7	0.0

### Educational kit, mentoring and peer group support

Reactions varied concerning the use of the kit: some coordinators reported that the kit was beneficial for planning activities and programs or provided useful material for distribution. For others, the kit was less helpful, mainly due to lack of time for material review or finding the content to be less relevant to their targeted population’s needs. Of those surveyed, 53% of coordinators reported frequent use of the kit, 20% reported occasional use of the kit and 27% did not use the kit at all.

The social media portal consultation group led to many discussions. The main topics included recommendations for publicizing activities, requests for assistance, recommendations for suppliers, feedback after collaboration with suppliers and advice from the research team. Participants reported that the social media portal was an effective platform for peer consultation. The participating DMCCs planned and implemented multifaceted HP initiatives throughout the intervention, presented in Supplemental Table 2.

### EQUIHP evaluation

**Fig. 2:**
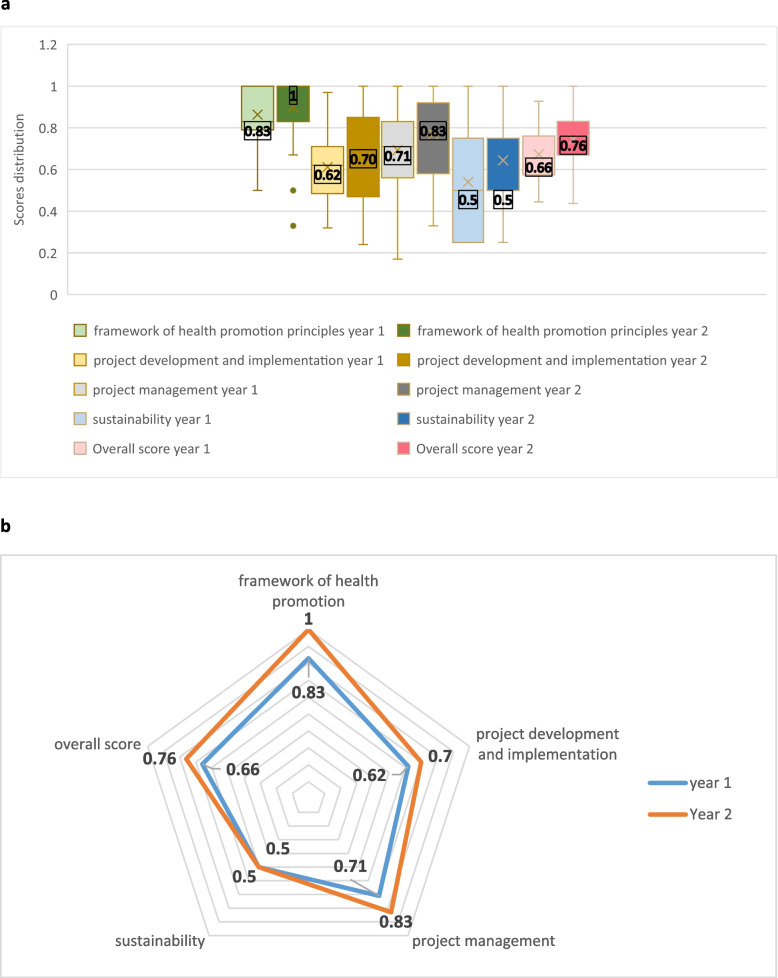
**a** Results from EQUIHP evaluation of health initiatives in years 1 and 2. **b** Spider chart comparison of the 2 years EQUIHP evaluation. Legend: Median scores per domain and distribution [0–1 range]

The results of the EQUIHP evaluation of HP initiatives during years 1 and 2 are presented in Fig. 2a and b. A total of 38 HP initiatives were reported in year 1 and 66 in year 2, with a participation of approximately 29,191 residents.

Our findings show an improvement in all domains during the second year, with a total median score of 0.76 in year 2 compared to 0.66 in year 1. The ‘Framework of HP principles’ domain received the highest median score in both years (0.83 and 1.0, respectively). The ‘Project development and implementation’ received median scores of 0.62 and 0.70, with a broader range in year 2 (IQR _year1_ = [0.20] and IQR_year2_ = [0.38]). The ‘Project management’ domain presents a high concentration of scores observed in the upper level in the two years (median scores of 0.71 and 0.83) with a more marked lack of symmetry in Year 2. The ‘Sustainability’ domain presents the lowest scores in both years. It remains stable (median score of 0.5), without improvement in Year 2 but with a lower extent of dispersion (IQR _year1_ = [0.31], IQR_year2_ = [0.25]).

### Final RE-AIM evaluation

The results of the RE-AIM evaluation and overall scores of each DMCC are presented in Table 4. The scores are varied and range from 0.24 to 0.97. Six DMCCs (30%) were high performers, with a score in the [0.66–1] range. Most DMCCs (*n* = 13, 65%) were mid-range performers (within the [0.33–0.66] range), and only one (5%) was a low performer ([0–0.33] range).

**Fig. 3:**
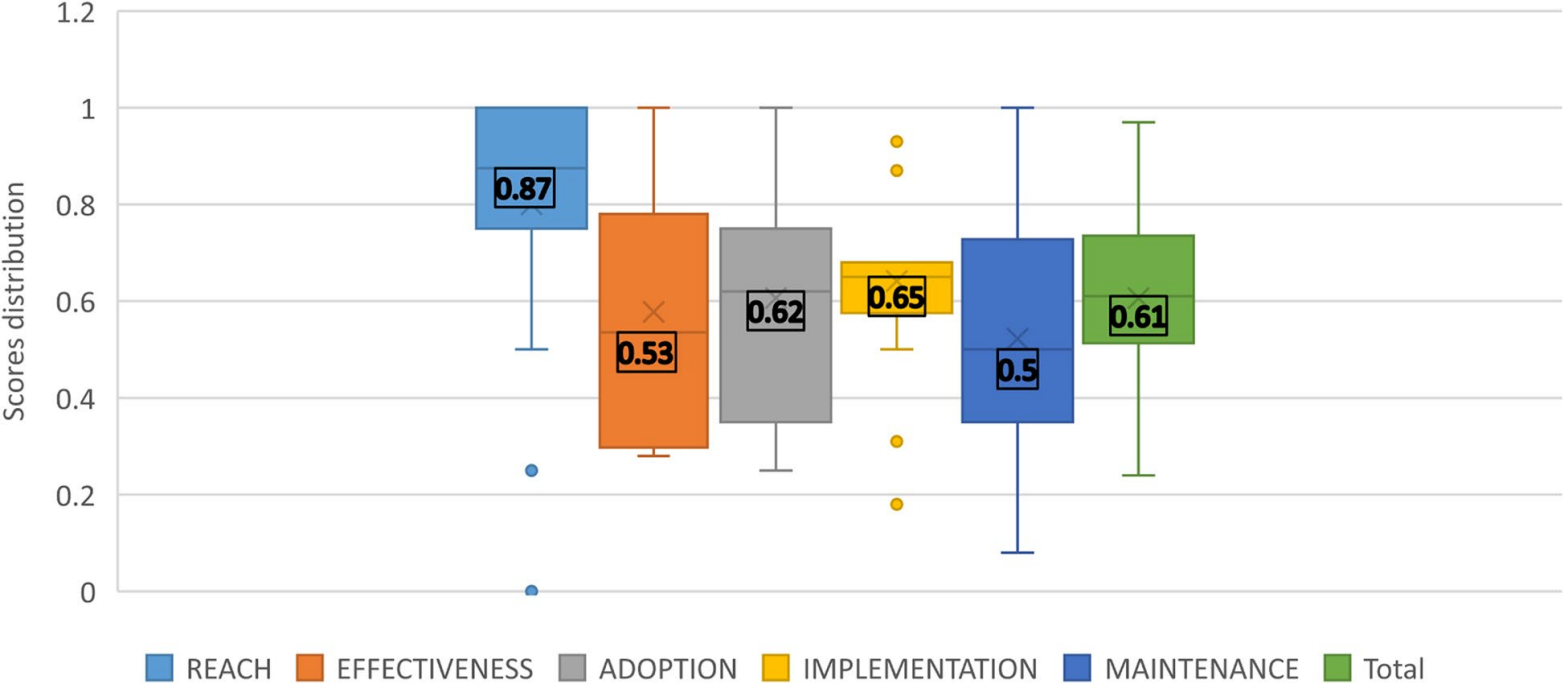
Results from RE-AIM evaluation of DMCCs at the end of year 2. Legend: Median scores per domain and distribution [0–1 range]

Median scores are presented in Fig. 3. Our findings show a total median score of 0.61, for all domains and DMCCs. The Reach component received the highest median score (0.87), with high scores for most DMCCs (17 DMCCs with a score above 0.66). The Effectiveness component ranged broadly, with a median score of 0.53. The Adoption component distribution range presents a high concentration of scores in the upper level, and a median score of 0.62. The Implementation component presents a median score of 0.65, with a concentration of scores in the mid-high level and low extent of distribution. The Maintenance component received the lowest median score (0.5), with a relatively large extent of distribution.

**Table 4:** Results from RE-AIM evaluation of participating DMCCs at the end of year 2

District Community Municipal Center ID	Reach	Effectiveness	Adoption	Implementation	Maintenance	Overall score
**1**	0.75	1	0.75	0.93	0.5	0.81
**2**	1	0.57	0.66	0.68	0.91	0.72
**3**	1	0.85	0.25	0.62	0.08	0.52
**4**	0.75	0.28	1	0.87	0.58	0.69
**5**	0	0.28	0.33	0.31	0.08	0.24
**6**	0.75	0.5	1	0.5	0.5	0.62
**7**	1	0.71	0.75	0.68	0.91	0.79
**8**	1	0.42	0.66	0.68	0.66	0.64
**9**	1	0.28	0.33	0.68	0.58	0.52
**10**	1	0.64	0.83	0.68	0.83	0.76
**11**	0.75	0.78	0.33	0.56	0.08	0.48
**12**	1	0.78	0.41	0.62	0.41	0.60
**13**	0.75	0.35	0.5	0.62	0.5	0.52
**14**	0.75	0.28	0.41	0.62	0.41	0.47
**15**	1	0.92	0.75	0.87	0.33	0.74
**16**	0.75	0.5	0.58	0.62	0.33	0.52
**17**	0.25	0.42	0.33	0.18	0.5	0.34
**18**	1	0.71	0.5	0.68	0.75	0.66
**19**	1	1	1	0.87	1	0.97
**20**	0.5	0.28	0.75	0.56	0.5	0.51

*Legend*: Absolute scores per domain [0-1 range]

## Discussion

This program aimed to increase and expand DMCCs’ HPPs as well as to establish a peer network between DMCCs, at the community level. The EQUIHP and RE-AIM evaluation found that in the setting of this intervention, most DMCCs planned and implemented HP initiatives that demonstrated at least partial adherence to HP principles and practices in both work plans and implementation. The DMCCs were interested in participating in the program, likely due to the programming budget available for those who participated. Throughout the two years of activity, the DMCCs developed many initiatives in HP, and efforts were made to reduce health gaps and communicate HP messages to residents and employees. HP activities within the community ranged from a one-time “health event” or lecture to integrated and long lasting programs for various target populations and with diverse health contents. Some DMCCs even generated policy changes within the DMCC (healthier refreshments, enabling physical activity time for employees, strengthening the communication with residents). The training seminar in the first year offered an opportunity for collaborations and interactions, and enabled the creation of a peer network of DMCCs to promote health in the urban setting. Participants in the training seminar reported that the seminar shifted their perception of other DMCCs from competitors to potential collaborators. In the second year, the creation of a social media portal group engendered sharing of successes and offered an effective platform for consultation regarding challenges, dilemmas or even suppliers’ details. Similarly, group meetings during the year enabled further connections. This peer network led to mutual assistance and facilitated exchange of information and documentation of experiences. The coordinators met colleagues and partners, operating in similar domains and with common goals.

### EQUIHP and RE-AIM domains

The evaluation findings emphasized the need to continue strengthening best practice interventions for health, based on HP knowledge and skills. Similarly, our findings emphasize the need to develop interventions that promote the sustainability of the HPPs.

The EQUIHP and RE-AIM based evaluations assessed all the DMCCs’ HP initiatives that evolved from program participation. These tools have been commonly applied to assess the impact of HP interventions in multiple domains [3, 14–18] and to enhance their quality, making possible the identification of best practice plans and strategies.

The ‘Framework of HP principles’ (EQUIHP) and ‘Reach’ (RE-AIM) domains received the highest median scores, whereas the ‘Sustainability’ (EQUIHP) and ‘Maintenance’ (RE-AIM) scores received the lowest. Most DMCCs had high scores in the ‘Reach’ domain, likely reflecting their use of existing communication infrastructures, which allowed for low cost, easy contact with residents. The high scores in the EQUIHP’s ‘Framework of HP principles’ domain, which measures inclusion, empowerment, and equity, indicated that the DMCCs effectively incorporated these HP principles into their health initiatives.

 Performance in other domains was more variable. DMCCs demonstrated a broad range of scores in the RE-AIM’s ‘Effectiveness’ domain, which assessed the use of best practices in problem definition (identifying health issues) and solution generation (developing a plan with defined objectives and strategies). These strategic planning components are essential for effective HP, as they provide practical tools for identifying priorities and designing relevant interventions [19–22]. Fawcett et al. found that assistance is often required for community initiatives in assessing needs and interpreting epidemiological data [23], suggesting that researchers should assist in identifying community health concerns and strategic planning.

The ‘adoption’ RE-AIM component was related to the degree of support that coordinators received from their director. The level of support accorded to HP programs within the setting, is one of the components of “Readiness to change”. Edwards and colleagues suggest that the effective implementation of a local program and support will be principally determined by this readiness for change [24].

In our study, the ‘Implementation’ RE-AIM domain assessed capacity building, as well as the ‘Project management’ EQUIHP domain. The mentoring, telephone calls and consultation group provided continuous feedback, possibly explaining these domains’ middle to high-level scores. This ‘open system’ of communication allowed for sharing of 'real world' solutions in vivo, likely improving implementation [25]. Other community-based programming studies [26] suggest that assistance from external consultants can facilitate the use of evidence-based practices, policy and systems change, as well as regional and cross-jurisdiction collaboration. Furthermore, several participants felt that direct technical assistance could play a key role in supporting regions to develop sustainability plans and secure long-term funding [26].

In the current study, the RE-AIM ‘Maintenance’ and EQUIHP ‘Sustainability’ domains obtained the lowest median scores. This is not surprising, as there is limited evidence that community interventions translate into sustained programs. One review found that approximately 40% of community-based organizations’ programs stop in their initial years of implementation, often following discontinuation of funding [27]. The current study’s HPCC program sought to pre-empt this common challenge by empowering local organizations to solve problems locally. Their efforts in planning and implementing HPPs in their community are likely to have the most sustainable impact in setting local norms and policy. As “leaders for change”, the coordinators may understand and disseminate the concept of HP and may affect the allocation of more resources for HP and public health. However, the need of internal and external resources, human and financial, presented many limitations for adoption and maintenance of HPPs within DMCCs. Additional factors influencing sustainability include the level of initiative acceptance within communities, long-term availability of resources including volunteers and funding, organizational stability, and the fluctuating political climate [27]. Longer term collaboration between researchers and communities may also influence sustainability of HPPs, involving mutual understanding and commitment [28]. High staff turnover in DMCCs, as was the case in the current study, may have compromised sustainability since the history of working together and building trust and capacity was less relevant for the program continuation.

### Innovative study components

The current study applied the healthy cities model to community centers, so as to facilitate locally-adapted HP programming. The Healthy Cities Model is used to promote city-wide health globally; however, it has not to our knowledge been applied on a smaller scale, within community centers. The current program integrated the Healthy Cities model into its HPCC training, encouraging coordinators to identify and address local health concerns, utilize local resources, and create a network both within and between communities. It presents an opportunity to establish healthy public policies at the local sub-municipal levels. Our program may provide a framework that supports and maintains health initiatives at the local level, enabling DMCCs to become health-promoting settings that lead to healthier communities and reduced health disparities. The collaboration and cooperation among DMCCs fostered by this program may lead to a broader community health vision, forging coalitions that can advocate more powerfully for HP. This new network helped focus on a common HP purpose, increased involvement and sharing, provided information about one another’s initiatives, and allowed participants to express and resolve doubts about planned activities**.** Indeed, the development of coalitions of community organizations to combat chronic health conditions can be seen as an intervention strategy [29–32].

This study also presents a pragmatic use of the EQUIHP and RE-AIM frameworks, as recommended in a 20-year review on RE-AIM framework application and evolution [33], and assessed factors described in the Stage Theory of Organizational Change model [34], including community empowerment and organizational change factors. Our evaluation included the essential factors for community empowerment and organizational change and presents how EQUIHP and RE-AIM frameworks can be used to address them. This emphasis on organizational-level and contextual factors for HP as best-practice strategic planning and resource identification, is also seen as an emerging area for RE-AIM [33]. Moreover, the pragmatic use of the EQUIHP and RE-AIM frameworks in this study can help identify specific factors essential for success in community settings.

The current program included training and mentoring as well as an educational kit, which allowed for increased assimilation of skills and access to HP materials. These aspects of the program presented a practical tool for effective HP [35, 36]. The effective implementation and maintenance of health initiatives among DMCCs not only requires motivated and involved coordinators, but also appropriate skills and HP capacity in order to operate an effective partnership and to be perceived as legitimate [37].

### Comparison with similar programs worldwide

While our HPCC program uniquely integrates the Healthy Cities model, training, and mentoring, there are several other community-based HP programs that had similar components. The Community Transformation Grants (CTG) program of the Center for Disease control (CDC), supported national networks of community-based organizations to implement evidence-based community preventive health programs targeting reduction in chronic disease and lessening of health disparities. In this program, they provided awardees with a list of recommended evidence and practice-based strategies that could be implemented, rather than the performance of a local needs-assessment and development of a local program based on needs. The YMCA community centers which participated in this program implemented several established programs [38–40]. CTG programs implemented at the state level enabled community-based tailoring of these interventions [26, 41]. This program was unfortunately terminated early, and outcomes data is limited. The CDC has similar programs targeting health disparities such as the REACH program [42], which provides funding and expert assistance to community-based organizations, academic centers and state and local health departments, but not to community centers. Several of their programs were evaluated, but the training and mentoring aspects of the program to our knowledge have not been assessed. Other studies have explored partnerships between health organizations and individual community centers so as to implement specific programs [43], and partnerships with community centers so as to recruit participants for interventions [44]. Compared with these studies, the current evaluation was more detailed and included all program components.

### Limitations

The current study was limited in that it was adapted to the unique structure of the Jerusalem municipality, wherein DMCCs do not solely provide classes and activities, but are actively involved in urban planning and creating neighborhood policy. The evaluation was based on self-report of the coordinators, and the reliability of reporting may have varied. The final evaluation relied on the interpretation of the investigators, with peer consultation when necessary. Additionally, health outcomes and behavior changes were not measured, as community-wide assessment was not feasible in this study.

## Conclusions

The program presented in this paper reached a majority of DMCCs and led to the implementation of multifaceted HPPs with high participation rates in diverse target populations, and the creation of a new HP network within community centers. DMCCs varied in their fulfillment of HP requirements, with highest scores in empowerment, equity, and reach and lowest scores in sustainability. Training local community center staff members to assess and address local health concerns and build community HP capacity led to the development of HPCCs within the larger municipal framework. The optimization of HPCCs is a logical extension of the Healthy Cities framework and presents an opportunity to establish healthy public policies at the local sub-municipal levels. The local focus and efforts may help community actors to create HPPs more likely to be adopted, feasible in the ‘real-world’ and able to impact public health in the communities where people live.

## Supplementary Information

**Additional file1: Appendix A. **“Community center for the promotion of good health” agreement. **Supplemental material table 1.** Curriculum outline of training seminar, year 1.  **Supplemental material table 2. **District municipal community centers’ health initiatives. 

## Data Availability

The data that support the findings of this study are available from Dr. Donna R Zwas but restrictions apply to the availability of these data, which were used under license for the current study, and so are not publicly available.

## Abbreviations

HP: Health promotion
WHO: World Health Organization
HPPs: Health Promotion Programs
DMCCs: District Municipal Community Centers
DMCC: District Municipal Community Center
JCH: Jerusalemites Choose Health
HPCC: Health-Promoting Community Center
HPCCs: Health-Promoting Community Centers

## References

[1] WHO. Ottawa charter for health promotion. 1986. https://www.who.int/teams/health-promotion/enhanced-wellbeing/first-global-conference. Accessed 10 Jun 2022.

[2] Tsouros AD (1991). World Health Organization Healthy Cities project : a project becomes a movement : review of progress 1987 to 1990.

[3] Besor O, Manor O, Paltiel O, Donchin M, Rauch O, Kaufman-Shriqui V (2020). A city-wide health promotion programme evaluation using EQUIHP: Jerusalem Community-Academic Partnership (J-CAP). Eur J Public Health.

[4] Jerusalem municipality- the official website. https://www.jerusalem.muni.il/en/. Accessed 10 Jun 2022.

[5] IACC Israel Association of Community Centers. https://www.matnasim.org.il/english. Accessed 6 Jun 2022.

[6] Bollars C, Kok H, Van den Broucke, S Molleman G. User Manual. Woerden: European Quality Instrument for Health Promotion (EQUIHP); 2005. https://ec.europa.eu/health/ph_projects/2003/action1/docs/2003_1_15_a11_en.pdf. Accessed 28 Sept 2022.

[7] Re-Aim Planning Tool. Re-Aim.Org. Accessed 10 Jun 2022.

[8] European Quality Instrument for Health Promotion (EQUIHP). http://ec.europa.eu/health/ph_projects/2003/action1/docs/2003_1_15_a10_en.pdf. Accessed 10 Jun 2022.

[9] Glasgow R, Vogt T, Boles S (1999). Evaluating the public health impact of health promotion interventions: the RE-AIM framework. Am J Public Health.

[10] Galaviz KI, Harden SM, Smith E, Blackman KCA, Berrey LM, Mama SK (2014). Physical activity promotion in Latin American populations: a systematic review on issues of internal and external validity. Int J Behav Nutr Phys Act.

[11] Glasgow RE, Harden SM, Gaglio B, Rabin B, Smith ML, Porter GC (2019). RE-AIM planning and evaluation framework: Adapting to new science and practice with a 20-year review. Front Public Health.

[12] Dawson Beth; Trapp RG. Basic & Clinical Biostatistics. 4th ed. New York: Lange Medical Books-McGraw-Hill, Medical Pub. Division, c2004; 2004.

[13] Cerdá-gómez R, Paredes-carbonell JJ, López-sánchez MP (2018). Aplicabilidad y percepción de utilidad del European Quality Instrument for Health Promotion ( EQUIHP ) en un programa de promoción de la salud. Gac Sanit..

[14] Welch A, Healy G, Straker L, Comans T, O’Leary S, Melloh M (2020). Process evaluation of a workplace-based health promotion and exercise cluster-randomised trial to increase productivity and reduce neck pain in office workers: A RE-AIM approach. BMC Public Health..

[15] Greenberg KL, Donchin M, Leiter E, Zwas DR (2021). Health ambassadors in the workplace: a health promotion intervention mobilizing middle managers and RE-AIM evaluation of outcomes. BMC Public Health..

[16] Tountas Y, Dimitrakaki C, Bollars C, Van Den Broucke S, Kok H, Molleman G (2007). Evaluating quality in health promotion: the EQUIHP. Arch Hell Med..

[17] Brace AM, Padilla HM, DeJoy DM, Wilson MG, Vandenberg RJ, Davis M (2015). Applying RE-AIM to the Evaluation of FUEL your life: a worksite translation of DPP. Health Promot Pract..

[18] Estabrook B, Zapka J, Lemon SC (2012). Evaluating the implementation of a hospital work-site obesity prevention intervention: Applying the re-aim framework. Health Promot Pract..

[19] Hicking-Woodison L. Planning health promotion programs. Emerg Nurse. 2017.

[20] Public Health Ontario. At A Glance: The six steps for planning a health promotion program. https://www.publichealthontario.ca/en/health-topics/public-health-practice/program-planning-evaluation/planning-programs. Accessed 28 Sept 2022.

[21] King L, Gill T, Allender S, Swinburn B (2011). Best practice principles for community-based obesity prevention: development, content and application. Obes Rev..

[22] Flynn MAT, McNeil DA, Maloff B, Mutasingwa D, Wu M, Ford C (2006). Reducing obesity and related chronic disease risk in children and youth: a synthesis of evidence with “best practice” recommendations. Obesity Reviews.

[23] Fawcett SB, Paine-Andrews A, Francisco VT, Schultz J, Richter KP, Berkley-Patton J (2001). Evaluating community initiatives for health and development. WHO Reg Publ Eur Ser..

[24] Edwards RW, Jumper-Thurman P, Plested BA, Oetting ER, Swanson L (2000). Community readiness: research to practice. J Community Psychol.

[25] Chalmers ML, Housemann RA, Wiggs I, Newcomb-Hagood L, Malone B, Brownson RC (2003). Process evaluation of a monitoring log system for community coalition activities: five-year results and lessons learned. Am J Heal Promot.

[26] Walkinshaw LP, Mason C, Allen CL, Vu T, Nandi P, Santiago PM (2015). Process evaluation of a regional public health model to reduce chronic disease through policy and systems changes, Washington State, 2010–2014. Prev Chronic Dis.

[27] Savaya R, Spiro S, Elran-Barak R (2008). Sustainability of Social Programs. Am J Eval..

[28] Swerissen H, Crisp BR (2004). The sustainability of health promotion interventions for different levels of social organization. Health Promot Int.

[29] Kim DH (2016). Emergency preparedness and the development of health care coalitions: a dynamic process. Nurs Clin North Am.

[30] Cormier S, Wargo M, Winslow W (2015). Transforming health care coalitions from hospitals to whole of community: Lessons learned from two large health care organizations. Disaster Med Public Health Prep..

[31] Khare MM, Núñez AE, James BF (2015). Coalition for a Healthier Community: Lessons learned and implications for future work. Eval Program Plann..

[32] Lengerich EJ, Kluhsman BC, Bencivenga M, Allen R, Miele MB, Farace E (2007). Development of community plans to enhance survivorship from colorectal cancer: community-based participatory research in rural communities. J Cancer Surviv..

[33] Glasgow RE, Harden SM, Gaglio B, Rabin B, Smith ML, Porter GC (2019). RE-AIM planning and evaluation framework: adapting to new science and practice with a 20-year review. Front Public Heal.

[34] Steckler A, Goodman R, Kegler M (2002). Mobilizing organizations for health enhancement. Health behavior and health education: theory, research, and practice.

[35] Feyissa GT, Balabanova D, Woldie M (2019). How effective are mentoring programs for improving health worker competence and institutional performance in africa? A systematic review of quantitative evidence. J Multidiscip Healthc.

[36] Ramos IN, May M, Ramos KS (2001). Environmental health training of promotoras in colonias along the Texas-Mexico border. Am J Public Health.

[37] Gray P, Senabe S, Naicker N, Kgalamono S, Yassi A, Spiegel JM (2019). Workplace-based organizational interventions promoting mental health and happiness among healthcare workers: a realist review. Int J Environ Res Public Health.

[38] Ackermann RT, Marrero DG (2007). Adapting the diabetes prevention program lifestyle intervention for delivery in the community: the YMCA model. Diabetes Educ.

[39] Faro JM, Arem H, Heston A-H, Hohman KH, Hodge H, Wang B (2020). A longitudinal implementation evaluation of a physical activity program for cancer survivors: LIVESTRONG® at the YMCA. Implement Sci Commun.

[40] Belza B, Petrescu-Prahova M, Kohn M, Miyawaki CE, Farren L, Kline G (2015). Adoption of evidence-based health promotion programs: perspectives of early adopters of Enhance®Fitness in YMCA-affiliated sites. Front Public Heal.

[41] Arcaya M, Reardon T, Vogel J, Andrews BK, Li W, Land T (2014). Tailoring community-based wellness initiatives with latent class analysis-massachusetts community transformation grant projects. Prev Chronic Dis.

[42] Division of Nutrition, Physical Activity, and Obesity, National Center for Chronic Disease Prevention and Health Promotion. https://www.cdc.gov/nccdphp/dnpao/state-local-programs/reach/index.htm. Accessed 10 Jun 2022.

[43] Yang CL, Bird ML, Eng JJ (2021). Implementation and evaluation of the Graded Repetitive Arm Supplementary Program (GRASP) for people with stroke in a real world community setting: Case report. Phys Ther.

[44] King AC, Campero MI, Sheats JL, Castro Sweet CM, Hauser ME, Garcia D (2020). Effects of counseling by peer human advisors vs computers to increase walking in underserved populations: the COMPASS randomized clinical trial. JAMA Intern Med.

