# Supplementary material for: Municipal community centers as healthy settings: evaluation of a real-world health promotion intervention in Jerusalem
Source: BMC Public Health. 2022 Oct 7;22:1870. doi: 10.1186/s12889-022-14220-7 (PMC9540711; doi:10.1186/s12889-022-14220-7)
Supplement: Supplementary file 1 — Additional file1: Appendix A. “Community center for the promotion of good health” agreement. Supplemental material table 1. Curriculum outline of training seminar, year 1. Supplemental material table 2. District municipal community centers’ health initiatives. [file 12889_2022_14220_MOESM1_ESM.docx]

**Additional material:**

Appendix A: "Community center for the promotion of good health" agreement

Supplemental material, Table 1: Curriculum outline of training seminar, year 1

Supplemental material, Table 2: District municipal community centers' health initiatives

**Appendix A**:


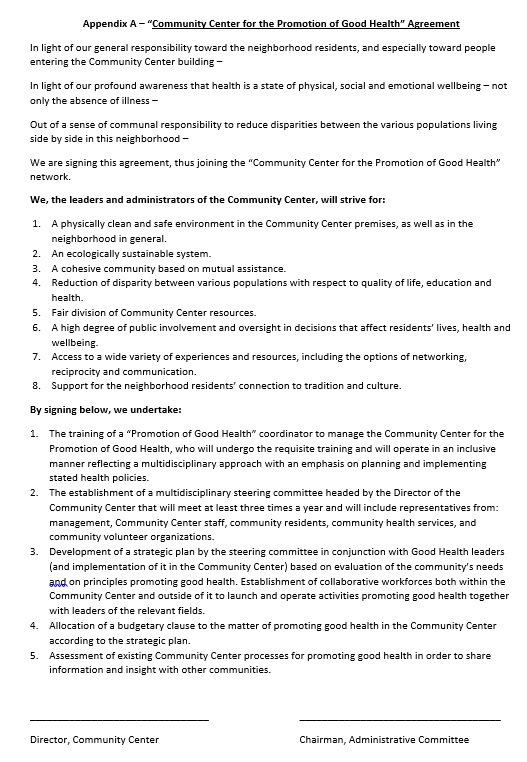


**Supplemental material, Table 1**: Curriculum outline of training seminar, year 1

| **Content** | **Session** |
| --- | --- |
| - Opening  - greetings , introductions , overview of the course - Healthy lifestyle survey and mapping questionnaire - Lecture on health promotion - Discussion on health promotion in our community center- What is happening today? - Matching expectations- individual and collective exercises | **Session 1** |
| - Field of knowledge - physical activity - Health-promoting settings at the national level - Discussion- from theory to practice - characteristics of our community - what exists in the neighborhood, healthy settings at the local level, mapping of existing health promoters, concerns and difficulties | **Session 2** |
| - Health promotion in different age groups- Focusing on the elderly - Community - background of different approaches in working with a community - Me and healthy lifestyle - Group discussions - Modeling and personal / community leadership | **Session 3** |
| - Program evaluation - measurement and assessment - Health promotion in different age groups- Focusing on early childhood - Initial needs assessment in my community | **Session 4** |
| - Nutrition - Smoking - The role of the community center in health promotion - workshop | **Session 5** |
| - HMOs - From principles to action - planning preliminary projects - Building a community project - principles for building and leading a community project, setting goals and objectives, budget, design thinking | **Session 6** |
| - Principles for building the project plan - Useful Tools for Coordinators - Dealing with objections, organizational values, budget, personal and community barriers , resources - Promotion of selected projects - group work ,  presentation of the projects | **Session 7** |
| - Branding , social networking, marketing - Panel of working and communities experienced in health promotion at the neighborhood and national levels - Collective work on the projects | **Session 8** |
| - Special populations - Building evaluation tools for selected projects – collective work - Health promotion skills , collective work on the projects, presentations , data collection in the field | **Session 9** |
| - Personal empowerment/ Developing personal skills - Meeting in one of the neighborhoods - presentation of an existing program , discussion with residents - Thinking out of the box - a creative session - Selection of best presentation | **Session 10** |
| - Principles for creating an active network as an alternative to traditional steering committees - Being a professional in the political tangle - Preparation of presentations and presentation of projects | **Session 11** |
| - Inspirational lecture - Principles and preservation of change , personal conclusions - Final exhibition of the projects , greetings , summary , certificates | **Session 12** |

**Supplemental material, Table 2:** DMCCs health initiatives

| Topic | Health Initiatives | DMCC ID |
| --- | --- | --- |
| Integrated initiatives for health | 1. Special "days for health", health topic days, healthy events 2. Healthy workshops 3. Early childhood health (sessions for women on nutrition, physical activity, hygiene and safety) 4. Lectures for seniors 5. Healthy summer camp 6. Playground for children "healthy land" 7. Raising awareness for health promotion among the DMCC's staff 8. Health promotion in the neighborhood (slogans and posters competition, postcards with health messages, articles on health in the neighborhood newspaper) 9. Health activities for the staff (healthy cooking, yoga, lectures) 10. Health club in the community 11. Healthy mothers and babies- sessions for women 12. Competition of the healthy family | 1, 3, 5, 7, 8, 13, 15, 20  1, 4  2  4, 18  5, 7, 8, 19  6  7  8, 13  10, 17  13  15  18 |
| Physical activity | 1. Physical activity for DMCC staff 2. Physical activity for all public 3. Physical activity for men only (fitness, coaching, training) 4. Physical activity for youth/ youth at risk 5. Physical activity for toddlers/ children 6. Physical activity for women (basketball team, training and empowerment, running groups, gymnastic classes, "walking strollers" and pilates for postpartum women, Zumba classes, activities for women 50+, Nordic walk, training at home) 7. Physical activity for people with special needs 8. Physical activity in public spaces 9. Physical activity for young families 10. Physical activity for seniors 11. Activities aimed to increase the number of participants in classes and number of active athletes in the neighborhood 12. Lectures on physical activity 13. The "stairs" competition (encouragement to take stairs) | 1, 2, 8, 15  19  1, 2  1, 8, 12, 15, 19  1, 14, 18, 19  4, 11, 13, 14, 15, 19  4  8, 10, 11, 14, 19  12  15, 19  3  3  11 |
| Nutrition | 1. Team empowerment - healthy recipe competition 2. Nutrition lectures (with a nutritionist, on food labelling…) 3. Nutrition/ healthy cooking workshops 4. Nutrition workshops for seniors 5. Nutrition activities for young girls 6. Nutrition activities for teenagers 7. Healthy diet- based on the Ethiopian culture 8. Nutrition of children- (lectures for parents, musical play for children) 9. Healthier refreshment options within the DMCC | 2  3  7, 14, 16, 18  3  8, 15, 18  12, 20  12  14, 18  18, 19 |
| Other | 1. Health topic day: safety and health 2. Lecture for Women on Breast Cancer 3. Alternative medicine 4. Herbal and natural pharmacy workshop 5. Dental hygiene 6. Residents' committee for cleanliness and sustainability of the neighborhood | 1  2  7, 20  14  18  7 |
